# The Impact of Sudden Public Health Events on the Insurance Companies' Investment Returns: Based on the Investors' Sentiment Perspective

**DOI:** 10.3389/fpubh.2022.810515

**Published:** 2022-02-15

**Authors:** Yunfeng Shang, Fangbin Qian, Nan Gao, Qin Yang, Yiting Guo, Yunpeng Sun

**Affiliations:** ^1^Northeast Asia Research Center, Zhejiang Yuexiu University of Foreign Languages, Shaoxing, China; ^2^Center for International Education, Philippine Christian University, Manila, Philippines; ^3^School of Hospitality Management, Zhejiang Yuexiu University of Foreign Languages, Shaoxing, China

**Keywords:** public health emergencies, insurance companies, investors' sentiment, COVID-19, stock portfolio returns

## Abstract

This study analyzes the conflicting effects of investors' sentiment caused by public health emergencies and uses event analysis methods and linear regressions to examine the impact of such emergencies on the stock prices of insurance companies. The study shows that public health emergencies have a positive and significant impact on insurance companies' portfolios through investors' sentiment, which is persistent. However, the investor fear index triggered by public health emergencies is negatively associated with insurance stock portfolio returns. Meanwhile, insurers with smaller market capitalization are more strongly influenced by investors' sentiment than those with larger market capitalization.

## Introduction

It is well documented that the pricing of certain assets is affected by people's emotions, which, in turn, are influenced by certain events, such as sunlight, moon phases, sporting events, and major aviation disasters. Argued that sunlight is a driver of people's moods and is positively related to daily stock returns. Yuan et al. in addition, international sporting events, especially soccer matches, can seriously affect investors' sentiment and, consequently, stock market investment returns. They also pointed out that major aviation disasters tend to generate negative sentiments within 2 days of the event. Moreover, many studies have shown that investors' sentiment may influence the institutional investors' investment decisions (1). Was the first to suggest that irrational emotions have a significant impact on the investors' actual financial market transactions and financial product choices, and emphasized the significant role of psychology in financial market behavior.

From the perspective of behavioral finance, some studies have demonstrated a significant relationship between the stock prices and investors' sentiment (2, 3). In particular, certain unexpected events may generate positive or negative emotions for different investors, leading directly to psychological biases and different investment expectations, thus affecting investors' actual investment decisions and stock market prices.

Sudden events, especially public health events, are likely to have serious and unpredictable socio-economic impacts. In the context of “Internet+,” public health emergencies have become a key topic of research for many scholars because of their rapid spread, wide scope, high hazard, and deep impact. A sudden public health event includes a major infectious disease epidemic, mass unexplained disease, major food poisoning, and other events that seriously affect public health (4). Major public health emergencies, such as SARS, Influenza A (H1N1), and Ebola, are viewed by the general public as events that generate tremendous negative sentiment and panic, but can create positive investor sentiment for certain institutions, such as the Wall Street, Morgan Stanley, and other institutional investors, which, in turn, can affect the stock prices of certain types of industries. Although the same unexpected public health event can generate a positive or negative sentiment, so far, there is little literature to explain the difference in the perception of the same event by different interest groups. Therefore, the novelty of this study is that it examines how the same public health emergency can have a devastating socioeconomic impact on the one hand, but on the other hand, the stakeholders in certain financial sectors, such as individual investors or institutional investors, may perceive this devastating event as a new investment opportunity for them, and institutional investors' sentiment is more affected than individual investors' sentiment (3).

This contradictory nature of investors' sentiment in the wake of a public health emergency is central to the era of “Internet+.” By combining online news media information with behavioral finance theories, the textual content of the online news media can be extracted more profoundly, and a media chain dominated by public opinion and emotions can be established to influence the actual investment behavior of different investors. Through an analysis of the extent of the use of positive and negative words in corporate news reports by online media, it can be found that investors use such online news media reports as reference content for investment decisions to guide their actual behaviors. Therefore, this study constructs new sentiment variables, namely disease-related news (DRNs), through official statements of the World Health Organization (WHO) and news media reports related to dangerous infectious diseases, which are used to examine the effect of investors' sentiment on the stock prices of the insurance companies.

Although public health emergencies spread fear and have a negative impact on public sentiment, the insurance companies take advantage of such public health outbreaks to promote and increase the sale of various insurance programs or insurance portfolio programs by marketing them as means to protect and fight against the deadly infectious diseases. This increase in publicity leads to irrational expectations on the part of investors, who start thinking that such policies will increase the cash flows of insurance companies, thereby giving a boost to their stock prices. Therefore, the main hypothesis of this study is that investors' fears induced by DRNs have a negative correlation with the stock prices of insurance companies; however, for some specific investors, DRNs have a positive impact on the stock prices of insurance companies.

In addition, after the outbreak of public health emergencies, the public pays extra attention to insurance products related to such diseases because of the panic psychology and risk aversion, which tends to increase the sales of insurance companies and the investment in the R&D cost of the corresponding products to a certain extent, making some investors feel optimistic about investing in such companies. Therefore, another hypothesis in this study is that some investors have a persistent positive sentiment toward the stock returns of insurance companies. Moreover, some scholars argue that the impact of investors' sentiment is more pronounced for smaller firms (5). Therefore, the final hypothesis of this study is that the smaller firms with relatively lesser market capitalization are more affected by investors' sentiment than the larger firms.

Based on the above analysis, to test the correctness of the hypothesis, this study uses the stock prices of 17 insurance companies listed on the Shanghai Stock Exchange, Shenzhen Stock Exchange, and Hong Kong Stock Exchange to construct three different types of stock portfolios, and the CSI 300 index is selected to test the robustness of the paper. In addition, two common empirical research methods, namely event analysis and multiple linear regression, are used to examine the impact of investors' sentiment generated by DRNs on the stock prices of insurance companies.

## Review of the Literature

### The Insurance Industry and the Investor Sentiment

The insurance industry has always been important for China's economy and society. After 2011, with the gradual regulation of the Internet in the insurance industry, a multi-layered protection system for Internet insurance was slowly realized. In 2015, when the usage of Internet in the insurance industry showed signs of growth, the China Banking and Insurance Regulatory Commission (CIRC) had clarified the responsibilities of the Internet insurance businesses, insurance institutions, self-owned online platforms, and third-party online platforms, and carried out industry further regulation of competition. From the perspective of personal insurance, health insurance and other protection-type insurance have high long-term growth potential. The penetration of Internet health insurance continues to increase, and it mainly relies on third-party channels. In the property insurance space, strict government regulations tend to limit the growth of Internet auto insurance to a certain extent, and auto insurance sales channels show a mobile trend. In the overall insurance industry, the high-quality Internet insurers have gradually become competitive. From the perspective of asset pricing, investors' sentiment is considered a non-systematic risk factor affecting stock prices. There are also many factors that influence investors' sentiment, such as weather conditions, sunshine hours in autumn and winter (6) and international soccer matches. Moreover, a large body of literature suggests that investors' sentiment has a significant impact on the stock market returns (2, 7). Stated that the confidence index has a significant positive impact on the stock market returns in the current period. Baker and Wurgler (2) argued that investors' sentiment has a significant effect on investors' behavior in terms of actual financial market decisions. For example, if an investor holds a pessimistic sentiment at the beginning of a stock investment, the future return of that stock will be higher. Moreover, it is suggested that there is a significant negative relationship between investors' sentiment and stock returns (7). In general, investors' fearful sentiment leads to a negative correlation between the returns of various assets, while a positive sentiment increases the investors' willingness to take risks. Despite the general public's negative sentiment due to the fear of being infected, the combination of public and societal demand to contain infectious diseases and to protect the assets of the insured population may generate significant additional revenue streams for the insurance companies. In response to public health outbreaks, the insurers may take effective measures, such as increasing human research and development expenditures for their insurance portfolio, publicity expenditures, and personnel training expenditures for different types of insurance policies for such diseases. Therefore, this paper focuses on the three major infectious diseases considered by the WHO as Public Health Emergencies of International Concern (PHEIC), namely the severe acute respiratory syndrome (SARS) outbreak in 2003, the Influenza A (H1N1) outbreak in 2009, and the Ebola outbreak in 2014 and 2018.

### Media Coverage

Vasterman et al. (5) suggested that the media can make a positive contribution to the prevention of infectious diseases by disseminating scientific methods of prevention and realistic information about the development of such diseases to reduce the overall level of anxiety among the general public about such infectious diseases. This has the effect of reducing public fear and maintaining social stability. However, some scholars have suggested that media may trigger excessive panic and overreaction among the public about the status of infectious diseases, which can directly lead to an imbalance between the actual and perceived risks (5). This overreaction and panic of the population can, to some extent, hinder economic development. For example, people may stay at home for long periods of time to reduce the likelihood of infection and reduce their travel time and access to public transportation, which can directly affect the revenue of the public transportation, food and beverages, and tourism industries and thus have a negative impact on the economy. For example, the SARS outbreak in 2003 cost China approximately 0.5% of its gross domestic product (GDP) (8) and the global economy between $30 billion and $100 billion (9).

To better understand the scale, timing, and importance of information salience of public health emergencies, based on the methodology of indicated that the frequency of news related to the official WHO PHEIC statement increases significantly on the day of the event, and the intensity of the news coverage increases over the following 3 days, reaching a peak. The findings suggest that the absolute number of news announcements remains above average for 6 days after the PHEIC announcement. This indicates, to some extent, that there is a linkage between the official news and media coverage, which has a constant effect on investors' sentiment.

In summary, DRNs may have two conflicting emotional effects simultaneously: the positive effect and the fear effect. For example, documented a negative and significant “war sentiment effect” and a positive and significant “holiday sentiment effect” in the 1973 Arab-Israeli war in Israel. First, the overemphasis of the media coverage on public health emergencies indirectly leads to a panic effect on the public and investors and can even trigger excessive anxiety, depression, and pessimistic selling attitudes among investors. Moreover, this sentiment of extreme anxiety and fear may spread to different sectors and lead to panic selling in the stock market, which, in turn, triggers a sharp decline in stock prices. Therefore, in Hypothesis 1, this study assumes that the investor fear index induced by DRNs may lead to a decline in insurance stock returns.

**Hypothesis 1. DRNs may lead to low investor sentiment, which, in turn, negatively affects the returns of the insurance-based equity portfolios**.

Second, although DRNs may have a panic effect on the stock market, they may be perceived by some institutional investors as a lucrative investment opportunity for certain interest groups. Some investors believe that the occurrence of public health emergencies reignites the public's concern about the insurance industry, and more households may invest their idle funds in insurance programs from the perspective of risk aversion, which will help improve the profitability of insurance companies. At the same time, to better meet the needs of insurance allocations of families, the insurance companies will further increase the setting of different insurance product portfolios, which will increase their labor costs and investment in research and development (R&D). Continuous investment in R&D costs will generate positive investor sentiment, which, in turn, will increase the demand for insurance stocks and further enhance the overall valuation of insurance portfolios. Therefore, in Hypothesis 2, this study assumes that there is a positive relationship between DRNs and the returns of insurance stock portfolios.

**Hypothesis 2. DRNs have a positive impact on the return of the insurance equity portfolios**.

Suggested that investors' sentiment is stronger for the small-cap stocks than for the large-cap stocks. Based on this proposed view, the third hypothesis in this study is that investors' sentiment may be stronger for the smaller market capitalization insurance companies than for the larger market capitalization insurance companies. Edmans et al. (10) suggested that the shares of smaller companies are largely held by local investors, who tend to be more influenced by specific events. In addition, as the smaller insurance companies have inherent limitations of cash flows, they prefer to keep costs low to have decent profit margins in response to public health emergencies. Therefore, when it comes to developing their own product portfolio and investments, these companies tend to imbibe a “herding effect” and follow the larger insurance companies. As a result, the investors are likely to pursue the smaller insurance companies because they think that these companies are more flexible in resource allocation, which can help them in generating a higher return for a lesser investment. Therefore, this study proposes hypothesis 3, which states that investors' sentiment driven by DRNs has a greater impact on the prices of insurance stocks of companies with smaller market capitalization than of companies with larger market capitalization.

**Hypothesis 3. DRNs have a greater impact on the stock returns of insurance companies with smaller market capitalization compared to insurance companies with larger market capitalization**.

In the early stages of a public health emergency, the public usually does not anticipate the development and spread of the event sufficiently, which may lead to underreaction for some investors. However, as news coverage of the event continues to be broadcast and the disease becomes more serious, some investors may overreact. The degree of investor overreaction ultimately depends on the relative significance of the information in the announcements reported by the media. The outbreak of a sudden public health event is bound to be over-interpreted by a significant number of investors, which, in turn, affects the market valuation of the industries related to the event, and the insurance industry is no exception to this general rule. Therefore, even if the insurance industry is not directly related to an event, the media can indirectly create a significant impact on its stock prices through an uninterrupted broadcast of news. Therefore, the following Hypothesis 4 is proposed in this paper.

**Hypothesis 4. DRNs have a persistent effect on the return of insurance stock portfolios due to the influence of relevant media reports**.

## Data and Research Methodology

This section describes the collection process of DRNs, the construction method of insurance class portfolios, and descriptive statistics. In addition, two commonly used empirical research methods, that is, event analysis and multiple linear regression, are used to verify the impact of DRNs on the insurance class portfolio returns. Due to the wide variety of public health emergencies, this paper only focuses on the major infectious disease categories as data research objects. By considering the data of the past 20 years, the top 10 major infectious diseases are summarized and outlined (as shown in Table 1). Among them, SARS, Influenza A (H1N1), Ebola in West Africa, and Ebola in the Democratic Republic of the Congo (DRC) were declared as PHEIC events by the WHO. The data in this paper cover the entire period considered as a PHEIC event by the WHO, that is, from February 1, 2003 to December 31, 2018. Furthermore, the scope of the data includes the major infectious diseases, that is, SARS, Influenza A (H1N1), and Ebola, covering 153 DRNs (see Appendix 1). In this study, this part of DRNs is studied and analyzed as event days.

**Table 1:** Global timeline of the top 10 major infectious disease related conditions.

**No**.	**Global infectious disease**	**Outbreak country**	**Outbreak time**
1	Pneumonic Plague	India	August, 1994
2	SARS	China	February, 2003
3	Avian Flu	China	February, 2006
4	Influenza A (H1N1)	The United States	April, 2009
5	MERS	Saudi Arabia	June, 2012
		Korea	June, 2015
6	Ebola	Guinea, West Africa and other countries	March, 2014
7	Zika Virus	Brazil	May, 2015
8	Cholera	Tanzania	August, 2015
9	Measles/Rubeola	Brazil	February, 2018
10	Ebola	DRC	August, 2018

*Data source: Guotai Junan Securities Research, CEIC, and WHO*.

As shown in Appendix 1, this paper classifies major events into the following categories: WHO statements, WHO outbreak news, approvals, research grants, and announcements. A WHO statement is a document that is obtained directly from the official WHO website and is formally announced by the WHO. It is a statement made to the public in the official capacity of the WHO that communicates all substantive news about the infectious disease in question. For example, on March 12, 2003, the WHO made an official statement and issued a global warning, which was followed immediately by another related health warning from the U.S. Centers for Disease Control and Prevention, illustrating the importance of the WHO statement and the level of attention it received worldwide. Moreover, the WHO recommended isolation and treatment of suspected cases and the establishment of a network of health care workers to assist in the study of the SARS outbreak. As a result, many media outlets use these WHO statements as a basis for disseminating news to the public.

The WHO outbreak news is information obtained from the official WHO website that describes the progress of the infectious disease in question and, to some extent, provides regular updates on the current situation. For example, for the Influenza A (H1N1) outbreak in the United States in April 2009, there were 43 WHO outbreak news articles within a 6-month period; every week, there was an official WHO news report on the outbreak. Moreover, 29 countries were affected by SARS during the outbreak, while the spread of Influenza A (H1N1) was even more alarming, with more than 210 countries involved in official announcements by August 2010, creating a global epidemic situation. The rapid spread of infectious diseases and the regular updates of WHO disease outbreak news are bound to attract the attention of capital market investors, which, in turn, affects investors' sentiment and capital market stock valuations.

In addition to the WHO statement and WHO outbreak news, major events categories include approvals, research funding, and announcements. Approvals relate to the government approvals for the development of new vaccines related to the company. Research funding is the official WHO announcement and grant of policy funding for the development and production of new vaccines, with the date of official funding determined as the date of research funding. Statements are lower-level announcements than WHO statements and include influential statements from relevant government agencies and companies regarding the current status of infectious diseases. All events are obtained through a rigorous online news search, and all relevant DRNs' announcements are categorized, summarized, and listed in Appendix 1.

### Portfolio Construction

To better examine the impact of DRNs on the stock prices of insurance companies, this study considers a total of seven insurance companies listed on the Shanghai Stock Exchange, Shenzhen Stock Exchange, and 10 insurance companies listed on the Hong Kong Stock Exchange, as shown in Appendix 2.

All the data are obtained from the Wind database in the classification of insurance in non-banking finance belonging to Shenyin Wanguo.

As the event date is from February 1, 2003 to December 31, 2018, the daily stock returns of 17 insurance listed companies from February 2003 to December 2018 are selected as data samples in this study. Moreover, three portfolios of insurance stocks are constructed in this study. The first one is the equally weighted insurance portfolio (EW), which can assess the overall returns of the insurance companies. In Table 2, this study sorts the 17 insurance stocks in order from the highest to the lowest total market capitalization as of December 31, 2018. Further, it divides these stocks into two types of portfolios according to their market capitalization. The second portfolio is the weighted portfolio of the top 10 insurance stocks by market capitalization (TOP), which shows the weighted market capitalization of the stocks most prominent in the insurance industry. The third portfolio is the weighted portfolio of the remaining seven stocks, which have a comparatively smaller market capitalization (BOTTOM).

**Table 2:** Breakdown of the total market capitalization of insurance stocks as of December 31, 2018.

**Stock code**	**Stock short name**	**Total market capitalization 1** **[Trade date] 12-31-2018** **[Unit] $**
2318.HK	Ping An of China	1,208,577,298,335.6100
601318.SH	Ping An of China	1,058,955,428,801.6600
1299.HK	AIA	785,009,145,765.0000
2628.HK	China Life	608,404,325,590.5040
601628.SH	China Life	533,083,870,082.4000
2601.HK	China Taipa Insurance	274,337,969,357.4530
1339.HK	People's Insurance Group of China	245,449,210,275.6900
601601.SH	China Tai Bao	240,374,928,751.0000
601319.SH	China Renminbi Insurance	215,062,598,043.5600
2328.HK	China Property and Casualty Insurance	178,164,550,077.0300
1336.HK	Xinhua Insurance	132,695,953,455.0250
601336.SH	Xinhua Insurance	116,268,194,417.2930
0966.HK	China Taiping	77,271,398,567.0000
1508.HK	China Reinsurance	67,967,692,936.0000
6060.HK	Zhong An Online	36,818,813,145.0000
000627.SZ	Tianmao Group	27,716,929,615.6500
600291.SH	Westwater	11,247,632,449.6200

*Data source: Wind database*.

### Descriptive Statistics

By calculating the daily stock returns for the three types of insurance stock portfolios described above from February 2003 to December 2020, a total of 6,544 observations are generated. Table 3 presents the descriptive statistics for these three types of portfolios. During the sample period, the average return of all three types of portfolios, EW, TOP, and BOTTOM, is negative, and the average return of the BOTTOM portfolio is −0.00885%, which is higher than the average return of the EW and TOP portfolios. The TOP portfolio has the highest variation between the maximum and minimum values, while the EW portfolio has the lowest variation. By comparing the values of the standard deviation, it can be concluded that the TOP portfolio has the highest unsystematic risk (10.98%); however, the BOTTOM portfolio has the lowest standard deviation (6.45%).

**Table 3:** Descriptive statistics of the three insurance-based stock portfolios.

	**EW**	**TOP**	**BOTTOM**
Mean	−0.012784	−0.015301	−0.008815
Median	0.000201	0.000000	0.000234
Maximum	0.117143	0.124050	0.182057
Minimum	−0.684513	−1.000000	−0.673271
Std. Dev.	0.088149	0.109782	0.064571
Skewness	−6.000809	−6.742884	−5.613968
Kurtosis	39.59130	51.20875	39.16907
Jarque-Bera	238262.6	402622.5	230439.1
Probability	0.000000	0.000000	0.000000
Sum	−49.29653	−59.00120	−33.99225
Sum Sq. Dev.	29.95431	46.46054	16.07311
Observations	3,856	3,856	3,856

*Data source: Eviews statistics*.

### Empirical Study

#### Event Analysis Approach

This study first investigates the impact of the average cumulative excess return (CAR) of DRNs on the price of insurance stocks through an event analysis approach. The excess return (AR) is the difference between the actual return of an insurance class stock portfolio and its expected return over the course of the entire event window, and CAR is the accumulation of excess returns over a specified time period. Generally, CAR is considered an important indicator to evaluate the increase in the shareholder value. In this study, the release date of the relevant infectious disease searched by the official news of the WHO and other online platforms is selected as the date of the event. In addition, (−10, 10) is selected as the event window period, that is, 10 days before and 10 days after each DRNs event date. Assuming that there is a stable linear relationship between the individual stock returns and market returns, this study adopts the market model approach, that is, the capital asset pricing model (CAPM), to estimate the expected return of the portfolio. This approach avoids the effects associated with stock market volatility and aims to reduce the spread of prognosis.

**Figure 1:**
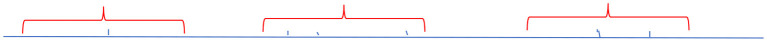
DRNs event selection criteria—“first event” approach.

Due to the multiple characteristics of infectious diseases, such as rapid spread, wide range, and complexity, there are numerous related news reports in a short period of time, which poses new requirements for this paper in terms of the method of selecting the date of the occurrence of the same type of news event. If all DRNs are considered within the event analysis method, the CAR faces the impact of overlapping event windows. To solve the problem of multiple occurrences of repetitive news reports within a short period of time, this paper now uses the “first event” as the criterion for selecting DRNs, that is, after the first DRN appears, all DRNs appearing within 20 days are ignored, and only the impact of CARs generated by the first event is focused on, and so on until the screening of the entire sample of DRNs is completed. For example, as shown in Figure 1, suppose there are six DRNs occurring on the date. According to the criteria of the “first event” in this paper to filter the occurrence date of DRNs, that is, to select and as the occurrence date of DRNs.

#### The Linear Regression Approach

This study follows the existing empirical studies (7, 10) to test the effect of DRNs on the insurance class stock returns. Therefore, the following regression model is constructed in this study:


(1) $$\begin{array}{l} R_{p,t}=\beta_{0}+\overset{5}{\underset{i=1}{\sum }}\beta_{1,i}R_{p,t-1}+\beta_{2}D_{t}+\beta_{3}E_{t}+\beta_{4}VIX_{t}+\xi_{it} \end{array}$$

In equation (1), *R*_*p, t*_ is the daily return of the insurance class stock portfolio p at moment *t*, β_0_ is the intercept, *R*_*p, t*−1_ is the daily return of the insurance class stock portfolio p at moment t-i, *D*_*t*_ is the dummy variable for the number of weeks, *E*_*t*_ represents the dependent variable of DRNs, *VIX*_*t*_ represents the investor fear index, which reflects the degree of investor pessimism about securities investment, and ξ_*it*_ is the error term.

As most DRNs selected in this study are from the official reports released by the WHO, there are time zone differences in the impact of DRNs on the Chinese stock market. This study assumes that the effect on the Chinese stock market occurs from the date of the release of DRNs. The returns of the previous days of each portfolio are also used as independent variables to ensure the correlation of all series. In addition, dummy variables are used to measure the “Monday effect,” which is common in the stock market. This study draws on the market volatility index proposed by and the S&P 500 Volatility Index (VIX), which measures the volatility of stock market prices. Generally speaking, when the VIX index is below 20, it indicates that the investors are positive and optimistic about the market, and the market performance will be relatively stable; when the VIX index is above 20, it indicates that the investors are divided about the current market scenario, which will cause significant market volatility to some extent. When the VIX index is below 15, it indicates that the investors are extremely optimistic about the market, and the market may appear irrationally positive. But when the VIX index is above 40, it means that the investors are extremely pessimistic about the market, and the market may appear to be in the grip of irrational panic, causing it to fall sharply.

## Empirical Results and Discussion

### Event Study Methodology

**Figure 2:**
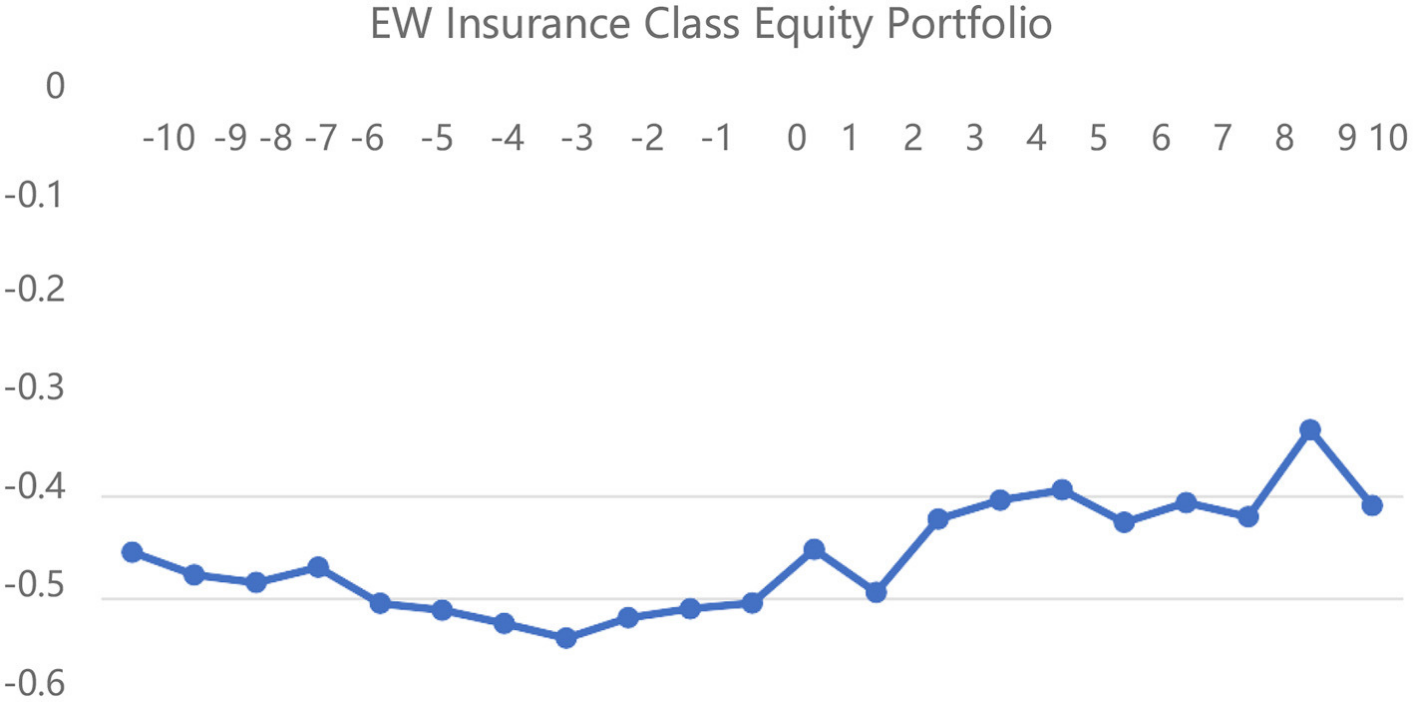
EW insurance equity portfolio cumulative excess return line chart.

**Figure 3:**
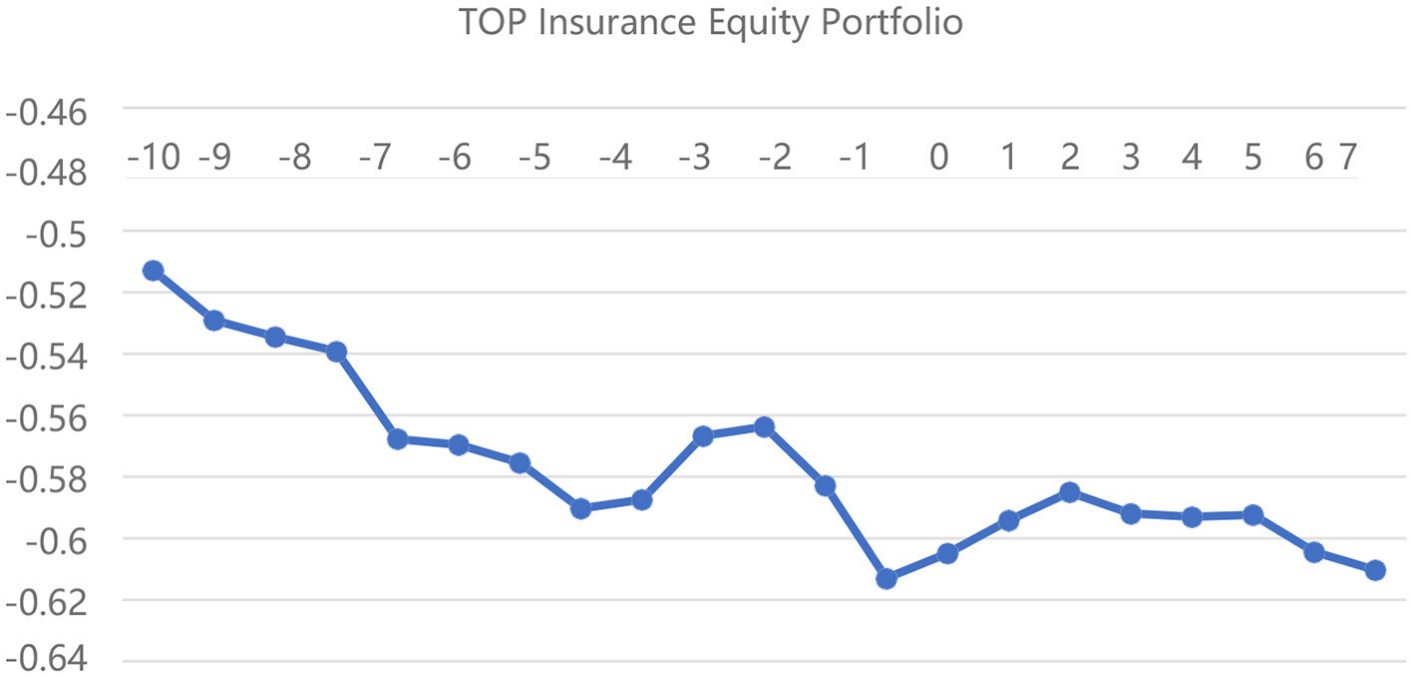
Cumulative excess return of the TOP insurance equity portfolio on a line chart.

**Figure 4:**
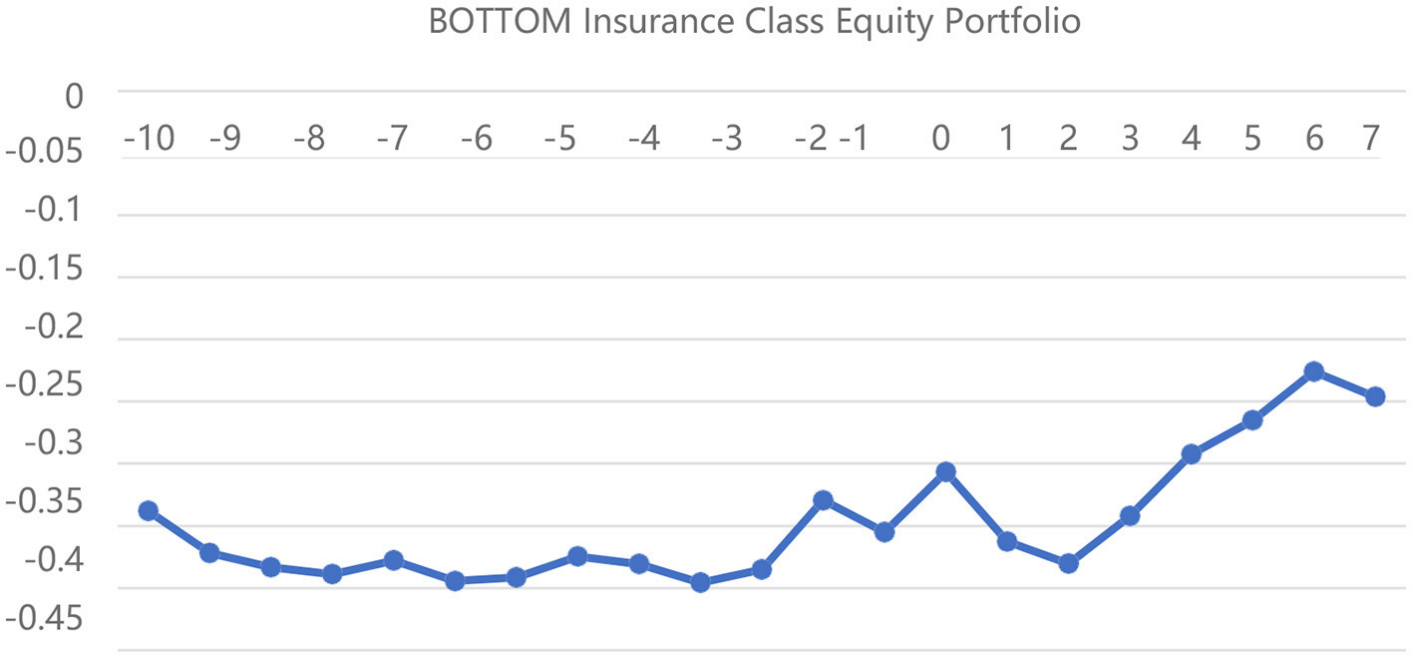
BOTTOM insurance equity portfolio cumulative excess return line chart.

Figures 2–4 depict the CARs of the different insurance portfolios based on the “first event” criteria for the pre- and post-event DRNs, respectively. First, the CARs of the three insurance portfolios remain negative throughout the window, indicating that the actual returns of the insurance portfolios are much lower than the expected returns, which also provides new trading strategies for different types of investors. Second, the returns of both EW and BOTTOM stock portfolios increase incrementally on the day of the DRNs event, and this persistent increase remains unchanged for 10 days afterward, which confirms the validity of Hypothesis 4 that DRNs have a persistent effect on insurance stock portfolio returns. Third, comparing Figures 3, 4, we can see that the CAR of the BOTTOM insurance portfolio is much higher than that of the TOP portfolio after the DRNs event date, mainly because the investors, who hold small-cap insurance stocks have a more positive sentiment, which drives the stock returns higher, proving the validity of Hypothesis 3. Overall, the event analysis method shows that DRNs have a persistent effect on the insurance stock returns and more positive investor sentiment for smaller-capitalization insurance stocks. However, due to a significant time horizon and subjective criteria for classifying DRNs, only 42 DRNs are selected to represent the impact of 153 DRNs on the insurance portfolio returns based on the “first event” criterion. Therefore, the results of the event analysis method are much weaker than those of the linear regression method.

### Linear Regression Method

Table 4 shows the regression results based on all DRNs on the stock returns of insurance companies. As shown in Table 4, the linear regression results for all three portfolios show a significant negative impact of the investor fear index (VIX), that is, there is a negative relationship between the investor fear index and insurance class stock returns, which verifies the validity of Hypothesis 1. This finding is consistent with the conclusion reached by, who stated that the investor fear index caused by the 1973 Arab-Israeli war had a negative impact on the stock market returns of the Tel Aviv Stock Exchange.

**Table 4:** Linear regression results based on all DRNs.

**Portfolio**									
EW	0.0149***	−0.0126**	−0.0253	0.0161	−0.0092	−0.0249	−0.0053	0.1726***	−0.0812***
	−0.0043	−0.0125	−0.2749	−0.1161	−0.1756	−0.2054	−0.1327	−0.0002	0
TOP	0.0221*	−0.0289	−0.0267**	−0.0205	−0.0049	−0.0321	−0.0073	0.1508**	−0.0635***
	−0.0537	−0.1641	−0.0148	−0.2437	−0.3873	−0.1547	−0.2079	−0.0175	0
BOTTOM	0.0716***	−0.0078	−0.0211	−0.0055	−0.0014***	−0.011	−0.0038***	0.2197***	−0.1005***
	−0.0032	−0.213	−0.2394	−0.3237	−0.0087	−0.3572	−0.0025	−0.0007	−0.0002

This table presents the results of the linear regression based on all DRNs, which cover the three portfolio categories (EW, TOP, and BOTTOM) of insurance stocks. This table uses the daily return data for the period from February 1, 2003 to December 31, 2020, involving 6,544 statistical values. The p-values are shown in parentheses. *represents significance at the 10% level. **represents significance at the 5% level. ***represents significance at the 1% level.

Second, as shown in Table 4, the coefficients of DRNs fluctuate from 0.1508 to 0.2653, and the *p*-values are all significant at the 5% level, indicating that all DRNs have a significant positive impact on the insurance equity portfolio, while verifying the correctness of Hypothesis 2. In the short term, the outbreak of such a contagion will have a certain degree of impact on the expansion of new policies in the life and property insurance industry and casualty insurance industry due to the restriction of offline marketing activities of the insurance agents, which, in turn, will limit the growth in new policy premium, thereby resulting in lesser growth in the insurance industry compared to the investors' expectations. However, in the medium to long term, the outbreak of such infectious diseases will once again stimulate the general public's awareness of insurance, especially health insurance, and increase the demand for insurance, which will have a positive effect on the development of health insurance. At the same time, it provides opportunities for the insurance companies to develop new types of protection products in response to the outbreak of infectious diseases, as well as, new growth points for their online business. In addition, on the basis of adjustable rates for the demand of long-term medical insurance, the government will further provide support for the innovation of new medical and health insurance products and develop a more flexible health insurance product portfolio. The outbreak of such infectious diseases may be seen as a new round of potential development opportunities in insurance development, along with an increase in the manpower-related costs and R&D investments of the insurers and government support. Thus, while the outbreaks of infectious diseases may have a non-negligible cost for national economies, DRNs can generate an optimistic and positive sentiment effect among investors and, in turn, influence the stock prices in the capital markets.

Third, although the insurance equity portfolio returns have been at negative return levels for a long time, they have shown an upward trend since the outbreak of the contagion. As shown in Table 4, the insurance portfolios with smaller market capitalization (BOTTOM) have higher investment returns than the portfolios with larger market capitalization. Moreover, as the coefficient of the investor fear index ranges from −0.0635 (TOP) to −0.1005 (BOTTOM), it indicates that investors' sentiment is stronger for the insurance companies with smaller market capitalization, but weaker for the insurance companies with larger market capitalization. This finding supports Hypothesis 3 that the returns of the insurance stock portfolios with smaller market capitalization are more strongly influenced by investors' sentiment than those with larger market capitalization, which is also consistent with and Edmans et al. (10).

Fourth, to test Hypothesis 4, this study uses six lagged returns of DRNs to capture the long-holding impact of DRNs on the insurance stock portfolios. Based on the previous findings of, 6 days after the PHEIC event, the number of media articles is still higher than the average number of media articles. In addition, noted that the announcements would typically make headlines a few days after the event. As the increase in the number of media articles will continue to inject a large amount of information into the capital market, this study assumes that investor optimism and positive investor sentiment will continue for six more days. As shown in Table 5, the coefficients of DRNs range from 0.4293 to 0.6871.

**Table 5:** Linear regression results based on all DRNs.

**Portfolio**									
EW	0.0137***	−0.0034**	−0.0197	0.0153	−0.0105	−0.0265	−0.0072	0.5386***	−0.1092***
	−0.0023	−0.0241	−0.5426	−0.1094	−0.2109	−0.3741	−0.1958	−0.0002	0
TOP	0.0109*	−0.0277	−0.0238**	−0.0278	−0.0073	−0.0372	−0.0087	0.4293**	−0.0769***
	−0.0587	−0.3672	−0.0105	−0.2658	−0.2674	−0.2544	−0.2659	−0.0003	0
BOTTOM	0.0659***	−0.0196	−0.0179	−0.0132	−0.0016***	−0.0241	−0.0056***	0.6871***	−0.0977***
	−0.0027	−0.2765	−0.2175	−0.2987	−0.0037	−0.3755	−0.0041	−0.0002	0

This table presents the results of the linear regressions based on all DRNs, which cover the three portfolio categories (EW, TOP, and BOTTOM) of insurance stocks. This table uses the daily return data for the period from February 1, 2003 to December 31, 2020, involving a total of 6,544 statistical values. The p-values are shown in parentheses. *represents significance at the 10% level. **represents significance at the 5% level. ***represents significance at the 1% level.

The coefficients of DRNs are significant at the 1% level, and the duration of positive investor sentiment is stronger for the insurance companies with smaller market capitalization, while the effect of positive investor sentiment is weaker for the insurance companies with larger market capitalization. Therefore, DRNs have a persistent effect on the return of the insurance stock class portfolio, and this finding verifies the validity of Hypothesis 4.

Finally, we verify the results of this study through robustness tests. First, the CSI 300 index is added as the fourth portfolio category to verify the findings of this study. As a cross-market index reflecting the overall trend of the Shanghai and Shenzhen markets, the CSI 300 index can better reflect the capital market conditions and evaluate the investment performance of companies, which is of great significance for studying the impact of investors' sentiment on the capital market through DRNs. Second, this study selects some DRNs instead of all DRNs to conduct multiple linear regressions on insurance stock portfolios. Finally, we re-run the regression model by changing the lag order in Equation 1. As shown in Table 6, through robustness testing, this study verifies the above four hypotheses and finds that investors' sentiment induced by DRNs has a negative impact not only on the insurance industry but also on the insurance stock portfolios. Further, it concludes that investors' sentiment may have a certain degree of impact on the valuation of the whole capital market, even at the global level.

**Table 6:** Linear regression results based on all DRNs.

**Portfolio**									
EW	0.0207***	−0.0227**	−0.0316	0.0287	−0.0085	−0.0274	−0.0046	0.1650***	−0.0651***
	−0.0021	−0.0283	−0.4565	−0.1785	−0.1549	−0.1976	−0.1563	−0.0001	0
TOP	0.0471*	−0.0306	−0.0399**	−0.0206	−0.0073	−0.0344	−0.005	0.1404**	−0.0548***
	−0.0682	−0.254	−0.0201	−0.3097	−0.2766	−0.1785	−0.1787	−0.0216	0
BOTTOM	0.0885***	−0.0139	−0.0265	−0.0116	−0.0023***	−0.0148	−0.0032***	0.2208***	−0.1650***
	−0.0039	−0.2659	−0.2584	−0.3452	−0.0056	−0.2315	−0.0019	−0.0005	0
沪深300	0.0581***	0.0098	−0.0652	−0.0086	−0.0031***	−0.0059	−0.0026***	0.2853***	−0.0263***
	(0.0044)	(0.2130)	(0.3076)	(0.2648)	(0.0091)	(0.3512)	(0.0015)	(0.0002)	(0.000)

This table presents the results of the linear regressions based on the selection of DRNs, which covers the three portfolios of insurance stocks (EW, TOP, and BOTTOM) and the CSI 300 index. The table uses the daily return data for the period from February 1, 2003 to December 31, 2020, involving a total of 6,544 statistical values. The p-values are shown in parentheses. *represents significance at the 10% level. **represents significance at the 5% level. ***represents significance at the 1% level.

## Conclusion

Based on the theory of behavioral finance, numerous studies have shown that the occurrence of specific events, such as war, disease, terrorism, and international sports competitions, inevitably has a significant impact on investors' sentiment. Therefore, this study tests the importance of investors' sentiment in the capital markets by examining the effect of DRNs on the stock prices of insurance portfolios. It is found that DRNs have a positive and significant impact on insurance class stock portfolios with persistent effects from February 2003 to December 2018, and this persistence is associated with the influx of media messages related to the category of DRNs into the market. In addition, the negative correlation between the fear index and insurance portfolio returns suggests that the influx of news about DRNs into the capital market causes some psychological pressure on investors and may generate negative investor sentiment and panic selling, which will directly lead to diminishing insurance portfolio returns. Moreover, the impact of DRNs on the smaller companies is greater than that on the larger companies in terms of market capitalization.

In general, DRNs can trigger both positive and pessimistic investor sentiment, which, in turn, can get combined in the capital market. Therefore, the findings of this study will support investors, especially institutional investors, fund managers, and financial analysts, in objectively viewing stock pricing and making proper investment decisions. The findings also show that in the context of DRNs, the insurance companies need to properly analyze their own business fundamentals and the strengths and weaknesses of their products to develop a product portfolio that suits the market demand.

## Data Availability Statement

The original contributions presented in the study are included in the article/Supplementary Material, further inquiries can be directed to the corresponding author/s.

## Author Contributions

Material preparation, data collection, and analysis were performed by YSh and YSu. The first draft of the manuscript was written by YSh. All authors contributed to the study conception and design, commented on previous versions of the manuscript, read, and approved the final manuscript.

## Funding

This work was supported by Northeast Asia Research Center, Zhejiang Yuexiu University funding project, Grant No. 2020DBY02.

## Conflict of Interest

The authors declare that the research was conducted in the absence of any commercial or financial relationships that could be construed as a potential conflict of interest.

## Publisher's Note

All claims expressed in this article are solely those of the authors and do not necessarily represent those of their affiliated organizations, or those of the publisher, the editors and the reviewers. Any product that may be evaluated in this article, or claim that may be made by its manufacturer, is not guaranteed or endorsed by the publisher.
