# Supplementary material for: The Impact of Sudden Public Health Events on the Insurance Companies' Investment Returns: Based on the Investors' Sentiment Perspective
Source: Front Public Health. 2022 Feb 15;10:810515. doi: 10.3389/fpubh.2022.810515 (PMC8885628; doi:10.3389/fpubh.2022.810515)
Supplement: Supplementary file 1 [file Table_1.docx]

Appendices

Appendix 1: Global Major Infectious Disease Event Days

| Date | Infectious Disease | Theme |
| --- | --- | --- |
| 03/12/2003 | SARS | WHO Statement |
| 03/17/2003 | SARS | WHO Statement, WHO Disease Outbreak News |
| 03/18/2003 | SARS | WHO Outbreak News |
| 03/19/2003 | SARS | WHO Outbreak News |
| 03/20/2003 | SARS | WHO Outbreak News |
| 03/21/2003 | SARS | WHO Outbreak News |
| 04/01/2003 | SARS | WHO Outbreak News |
| 04/03/2003 | SARS | WHO Outbreak News |
| 04/07/2003 | SARS | WHO Outbreak News |
| 04/10/2003 | SARS | WHO Outbreak News |
| 04/14/2003 | SARS | WHO Outbreak News |
| 04/15/2003 | SARS | WHO Outbreak News |
| 04/16/2003 | SARS | WHO Statement |
| 04/17/2003 | SARS | WHO Outbreak News, Statements |
| 04/21/2003 | SARS | WHO Outbreak News, Research Funding |
| 04/24/2003 | SARS | WHO Outbreak News |
| 04/28/2003 | SARS | Research Funding, Statements |
| 04/29/2003 | SARS | WHO Statement, WHO Outbreak News |
| 04/30/2003 | SARS | Statement |
| 05/01/2003 | SARS | WHO Outbreak News |
| 05/06/2003 | SARS | WHO Outbreak News |
| 05/09/2003 | SARS | WHO Outbreak News |
| 06/02/2003 | SARS | WHO Outbreak News |
| 06/26/2003 | SARS | Statement |
| 07/07/2003 | SARS | WHO Statement |
| 08/20/2003 | SARS | WHO Statement |
| 08/21/2003 | SARS | WHO Statement |
| 09/26/2003 | SARS | WHO Statement |
| 09/29/2003 | SARS | Research funding |
| 11/05/2003 | SARS | WHO Statement |
| 12/17/2003 | SARS | WHO Statement |
| 09/13/2004 | SARS | Research funding |
| 10/01/2004 | SARS | Research funding |
| 02/04/2005 | SARS | Research funding |
| 04/24/2009 | Influenza A (H1N1) | WHO Statement |
| 04/27/2009 | Influenza A (H1N1) | WHO Statement, WHO Disease Outbreak News |
| 04/28/2009 | Influenza A (H1N1) | WHO Disease Outbreak News |
| 04/29/2009 | Influenza A (H1N1) | WHO Statements, WHO Disease Outbreak News |
| 04/30/2009 | Influenza A (H1N1) | WHO Outbreak News |
| 05/01/2009 | Influenza A (H1N1) | WHO Outbreak News |
| 05/04/2009 | Influenza A (H1N1) | WHO Statement, WHO Outbreak News |
| 05/06/2009 | Influenza A (H1N1) | WHO Outbreak News |
| 05/07/2009 | Influenza A (H1N1) | WHO Statement, WHO Outbreak News |
| 05/08/2009 | Influenza A (H1N1) | WHO Outbreak News |
| 05/11/2009 | Influenza A (H1N1) | WHO Outbreak News |
| 05/13/2009 | Influenza A (H1N1) | WHO Outbreak News |
| 05/15/2009 | Influenza A (H1N1) | Statements |
| 05/18/2009 | Influenza A (H1N1) | WHO Outbreak News |
| 05/20/2009 | Influenza A (H1N1) | WHO Outbreak News |
| 05/22/2009 | Influenza A (H1N1) | WHO Outbreak News |
| 05/25/2009 | Influenza A (H1N1) | WHO Outbreak News |
| 05/27/2009 | Influenza A (H1N1) | WHO Outbreak News |
| 05/28/2009 | Influenza A (H1N1) | Statement |
| 05/29/2009 | Influenza A (H1N1) | WHO Statement |
| 06/01/2009 | Influenza A (H1N1) | WHO Outbreak News |
| 06/03/2009 | Influenza A (H1N1) | WHO Outbreak News |
| 06/08/2009 | Influenza A (H1N1) | WHO Outbreak News |
| 06/10/2009 | Influenza A (H1N1) | WHO Outbreak News |
| 06/11/2009 | Influenza A (H1N1) | WHO Statement |
| 06/15/2009 | Influenza A (H1N1) | WHO Outbreak News |
| 06/17/2009 | Influenza A (H1N1) | WHO Statement, WHO Outbreak News |
| 06/19/2009 | Influenza A (H1N1) | WHO Outbreak News, Statements |
| 06/22/2009 | Influenza A (H1N1) | WHO Outbreak News |
| 06/24/2009 | Influenza A (H1N1) | WHO Outbreak News |
| 06/25/2009 | Influenza A (H1N1) | Statement |
| 06/26/2009 | Influenza A (H1N1) | WHO Outbreak News |
| 06/29/2009 | Influenza A (H1N1) | WHO Outbreak News |
| 07/01/2009 | Influenza A (H1N1) | WHO Outbreak News |
| 07/03/2009 | Influenza A (H1N1) | WHO Outbreak News |
| 07/06/2009 | Influenza A (H1N1) | WHO Outbreak News |
| 07/08/2009 | Influenza A (H1N1) | WHO Statement |
| 07/13/2009 | Influenza A (H1N1) | WHO Statement |
| 07/15/2009 | Influenza A (H1N1) | Statement |
| 07/23/2009 | Influenza A (H1N1) | Statement |
| 07/24/2009 | Influenza A (H1N1) | WHO Statement |
| 07/27/2009 | Influenza A (H1N1) | WHO Disease Outbreak News |
| 07/31/2009 | Influenza A (H1N1) | WHO Statement |
| 08/04/2009 | Influenza A (H1N1) | WHO Disease Outbreak News |
| 08/06/2009 | Influenza A (H1N1) | WHO Statement |
| 08/12/2009 | Influenza A (H1N1) | WHO Outbreak News |
| 08/13/2009 | Influenza A (H1N1) | Statements |
| 08/21/2009 | Influenza A (H1N1) | WHO Statement, WHO Outbreak News |
| 08/28/2009 | Influenza A (H1N1) | WHO Statement, WHO Outbreak News |
| 09/04/2009 | Influenza A (H1N1) | WHO Outbreak News |
| 09/11/2009 | Influenza A (H1N1) | WHO Statement, WHO Outbreak News |
| 09/15/2009 | Influenza A (H1N1) | Approval |
| 09/18/2009 | Influenza A (H1N1) | WHO Statement, WHO Outbreak News |
| 09/24/2009 | Influenza A (H1N1) | WHO Statement |
| 09/30/2009 | Influenza A (H1N1) | Statement |
| 10/09/2009 | Influenza A (H1N1) | WHO Statement, WHO Outbreak News |
| 10/16/2009 | Influenza A (H1N1) | WHO Statement |
| 10/23/2009 | Influenza A (H1N1) | WHO Outbreak News |
| 10/30/2009 | Influenza A (H1N1) | WHO Statement |
| 11/05/2009 | Influenza A (H1N1) | WHO Statement |
| 11/06/2009 | Influenza A (H1N1) | WHO Outbreak News |
| 11/10/2009 | Influenza A (H1N1) | WHO Statement, Approval |
| 11/13/2009 | Influenza A (H1N1) | WHO Outbreak News |
| 11/19/2009 | Influenza A (H1N1) | WHO Statement |
| 11/20/2009 | Influenza A (H1N1) | WHO Statement |
| 11/27/2009 | Influenza A (H1N1) | WHO Outbreak News |
| 12/02/2009 | Influenza A (H1N1) | WHO Statement |
| 12/03/2009 | Influenza A (H1N1) | WHO Statement |
| 12/11/2009 | Influenza A (H1N1) | WHO Outbreak News |
| 12/22/2009 | Influenza A (H1N1) | WHO Statement |
| 01/07/2010 | Influenza A (H1N1) | Statement |
| 01/11/2010 | Influenza A (H1N1) | Statements |
| 01/22/2010 | Influenza A (H1N1) | WHO Statement, WHO Outbreak News |
| 02/05/2010 | Influenza A (H1N1) | WHO Outbreak News |
| 02/12/2010 | Influenza A (H1N1) | WHO Outbreak News |
| 02/26/2010 | Influenza A (H1N1) | WHO Outbreak News |
| 04/06/2010 | Influenza A (H1N1) | WHO Outbreak News |
| 04/16/2010 | Influenza A (H1N1) | WHO Outbreak News |
| 08/10/2010 | Influenza A (H1N1) | WHO Statement |
| 05/22/2012 | SARS | Research Funding |
| 03/24/2014 | Ebola | WHO Outbreak News |
| 07/18/2014 | Influenza A (H1N1) | Statements |
| 07/31/2009 | Influenza A (H1N1) | WHO Statement |
| 07/31/2014 | Ebola | WHO Disease Outbreak News |
| 08/06/2014 | Ebola | WHO Statement |
| 08/08/2014 | Ebola | WHO Statement |
| 08/11/2014 | Ebola | Statement |
| 08/12/2014 | Ebola | WHO Statement |
| 08/18/2014 | Ebola | WHO Statement |
| 08/27/2014 | Ebola | WHO Disease Outbreak News |
| 08/28/2014 | Ebola | WHO Statement |
| 09/01/2014 | Ebola | WHO Outbreak News |
| 09/05/2014 | Ebola | WHO Statement |
| 09/08/2014 | Ebola | WHO Outbreak News |
| 09/16/2014 | Ebola | WHO Statement |
| 10/01/2014 | Ebola | WHO Outbreak News |
| 10/09/2014 | Ebola | WHO Outbreak News |
| 10/28/2014 | Ebola | WHO Statement |
| 10/31/2014 | Ebola | WHO Outbreak News |
| 11/06/2014 | Ebola | WHO Statement |
| 05/08/2018 | Ebola | WHO Outbreak News |
| 05/11/2018 | Ebola | WHO Statement |
| 05/13/2018 | Ebola | WHO Statement |
| 05/16/2018 | Ebola | WHO Outbreak News |
| 05/18/2018 | Ebola | WHO Statement |
| 05/23/2018 | Ebola | WHO Outbreak News |
| 05/26/2018 | Ebola | WHO Outbreak News |
| 07/24/2018 | Ebola | WHO Statement |
| 08/02/2018 | Ebola | WHO Statement |
| 08/05/2018 | Ebola | WHO Outbreak News |
| 08/06/2018 | Ebola | WHO Outbreak News |
| 08/07/2018 | Ebola | WHO Statement |
| 08/14/2018 | Ebola | WHO Statement, WHO Outbreak News |
| 08/22/2018 | Ebola | WHO Outbreak News |
| 08/24/2018 | Ebola | WHO Outbreak News |
| 09/10/2018 | Ebola | WHO Statement |
| 09/23/2018 | Ebola | WHO Outbreak News |
| 09/25/2018 | Ebola | WHO Outbreak News |
| 10/20/2018 | Ebola | WHO Outbreak News |
| 12/27/2018 | Ebola | WHO Outbreak News |

Data source: The WHO’s official website, Wind database.

Appendix 2: Details of the insurance companies listed on the stock exchanges of Shanghai, Shenzhen, and Hong Kong

| Stock Code | Stock Short Name | Total Market Capitalization 1  [Trade Date] 12-31-2018  [Unit] Million |
| --- | --- | --- |
| 000627.SZ | Tianmao Group | 27,716.9296 |
| 600291.SH | Westwater | 11,247.6324 |
| 601318.SH | Ping An of China | 1,058,955.4288 |
| 601319.SH | PICC | 215,062.5980 |
| 601336.SH | Xinhua Insurance | 116,268.1944 |
| 601601.SH | China Tai Bao | 240,374.9288 |
| 601628.SH | China Life | 533,083.8701 |
| 0966.HK | China Taiping | 77,271.3986 |
| 1299.HK | AIA | 785,009.1458 |
| 1336.HK | Xinhua Insurance | 132,695.9535 |
| 1339.HK | People's Insurance Group of China | 245,449.2103 |
| 1508.HK | China Reinsurance | 67,967.6929 |
| 2318.HK | Ping An of China | 1,208,577.2983 |
| 2328.HK | China Property and Casualty Insurance | 178,164.5501 |
| 2601.HK | China Taipa Insurance | 274,337.9694 |
| 2628.HK | China Life | 608,404.3256 |
| 6060.HK | Zhong An Online | 36,818.8131 |

Data source: Wind database.
